# Physicochemically Tuned Myofibroblasts for Wound Healing Strategy

**DOI:** 10.1038/s41598-019-52523-9

**Published:** 2019-11-05

**Authors:** Ung Hyun Ko, Jongjin Choi, Jinseung Choung, Sunghwan Moon, Jennifer H. Shin

**Affiliations:** 1 Department of Mechanical Engineering, Korea Advanced Institute of Science and Technology, Daejeon, Republic of Korea; 2 School of Medicine, Konkuk University, Seoul, Republic of Korea; 3 BYON Co. Ltd., Seoul, Republic of Korea

**Keywords:** Biophysics, Cell biology, Therapeutics

## Abstract

Normal healing of skin wounds involves a complex interplay between many different cellular constituents, including keratinocytes, immune cells, fibroblasts, myofibroblasts, as well as extracellular matrices. Especially, fibroblasts play a critical role in regulating the immune response and matrix reconstruction by secreting many cytokines and matrix proteins. Myofibroblasts, which are differentiated form of fibroblasts, feature high cellular contractility and encourage the synthesis of matrix proteins to promote faster closure of the wounds. We focus on the functional characteristics of these myofibroblasts as the healing strategy for severe wounds where the surplus amount of matrix proteins could be beneficial for better regeneration. In this study, we first employed multiple physicochemical cues, namely topographical alignment, TGF-β1, and electrical field (EF), to induce differentiation of dermal fibroblasts into myofibroblasts, and to further activate the differentiated cells. We then used these cells in a mouse wound model to verify their potential as a transplantable substitute for the severe wound. Our results confirmed that physicochemically stimulated myofibroblasts promoted faster healing of the wound compared to the case with non-stimulated myofibroblasts through elevated matrix reconstruction in the mouse model. Conclusively, we propose the utilization of physicochemically tuned myofibroblasts as a novel strategy for promoting better healing of moderate to severe wounds.

## Introduction

Skin serves as the barrier against numerous pathogens, microorganisms, chemical toxins, and the other life-threatening sources in the environment. Therefore, any disruption of the barrier function of the skin, mostly due to cutaneous wounds, can lead to severe infections. Proper healing of the wounds, thus, is a critical physiological activity for self-protection of the body. Fortunately, for human, most of the injuries can heal naturally within a few weeks. For healthy healing of the wounds, the only clinical concern would be the rate and quality of regeneration. However, in the case of severe injuries, the volumetric loss would damage the dermal sites and exceed the natural regeneration capacity, which may be life threatening^1,2^. Acute burns, chronic ulcers, and traumatic accidents are marked examples of severe injuries where natural healing would be insufficient. The World Health Organization (WHO) announced that over 10 million suffer from burn-disability, and over 300,000 people die annually from burn-related cutaneous wounds^3^. Besides, the number of diabetes-related chronic wound complications has steadily increased^4–6^.

Scientific understanding of cutaneous regeneration process has led systematic and sophisticated technological advances in clinical wound repair strategies^7–13^. The vacuum-assisted closure (VAC), developed for chronic ulcers wound healing in the 1990s, has evolved into the widely known process called the negative-pressure wound therapy (NPWT)^14,15^. With the importance of delivery of cytokines at the wound site for proper healing^16^, the utilization of the negative pressure for enforcing the flow of body fluids at the wound site has been demonstrated to be effective^17^. This method, however, serves only as an auxiliary process to promote the natural healing of the wound, limiting its direct use for acute burns or other traumatic wounds. The skin graft, used for severe damages on regenerative sites, also has undergone significant improvement in both aesthetic and functional points of view^18–21^. Based on the physiological features of the wound site, researchers continue to optimize the thickness of the graft to minimize undesired complications and to meet the aesthetic and functional requirements^22^. Depending on the severity of the injury, either the full-thickness skin graft (FTSG) or the split-thickness skin graft (STSG) can be utilized^23^, but their applicability is limited by the shortage of donor tissue^24^. In addition to the repair strategies mentioned above, the cell-based therapies have been proposed as the promising alternative for all types of wounds regardless of the severity, donor availability, and other adverse effects^25–29^. Thus, this strategy has attracted a great deal of attention from many researchers in the field of cell and tissue engineering^30–32^. However, there remain many challenges to be addressed and investigated before going into clinical application^33–35^.

Natural wound healing process involves many different cell types, including epidermal keratinocytes, dermal fibroblasts, differentiated myofibroblasts, immune cells, and endothelial cells. Keratinocytes are critical in reepithelization for proper closure of the wound surface, and fibroblasts actively remodel matrix proteins to fill the damaged dermal tissue^36^. Thanks to their importance, these two cell types are most widely utilized for cell-based skin regeneration therapy^37–42^. During normal wound healing, fibroblasts transiently exhibit the activated phenotype of myofibroblasts which feature higher cellular contractility and produce excessive matrix proteins to promote the faster closure of the wound^43^. Despite its importance in matrix synthesis and tension production, the myofibroblasts have not been considered as a candidate for transplantation because these cells are often associated with scar formation. Under specific conditions, excessive synthesis of the matrix protein impairs healthy tissue healing, leading to pathological conditions such as hypertrophic scars, scleroderma, or Dupuytren’s disease^44^. For severely damaged tissue, however, the excessive production of matrix proteins may be useful, suggesting a potential utilization of myofibroblasts as candidate cells for wound therapy.

Growth factors play a significant role during the natural healing process. TGF-β1, generally known as a tension inducer in dermal tissue, induces differentiation of fibroblasts to myofibroblasts, thereby promoting matrix synthesis and inducing wound contraction^45^. Exogenous treatment of 10 ng/ml TGF-β1 on fibroblasts has been a gold standard for myofibroblast induction. In addition to the biochemical factors like TGF-β1, unique physical conditions can influence the activation and differentiation of the dermal fibroblasts^46,47^. For example, mimicking the polarized stretching of fibrous proteins during the natural healing process, culturing the cells on the patterned surfaces of aligned topography has been shown to enhance the differentiation capability of the fibroblasts when combined with TGF-β1^48^. The interstitial fluid, which can naturally occur due to swelling, high microvascular permeability, and increased lymphatic drainage, can also cause the collagen fiber alignment, thereby promoting the myofibroblasts differentiation^49^. While the phenotypic changes of fibroblasts by the uniaxially aligned topography have not been fully understood, the cellular elongation reinforced by the aligned pattern is known to stimulate cytoskeleton reorganization. The substrate stiffness also facilitates fibroblast-to-myofibroblast differentiation. According to Huang *et al*., MKL1-mediated mechanotransduction pathway regulates matrix stiffening, and the stiffened matrix induces differentiation of the lung myofibroblasts^50^. Besides, *in vitro* application of EF stimulation, otherwise naturally formed by ion leakage at the wound site, is effective in promoting the myofibroblast differentiation^51^.

Similarly to the natural healing process, these various physicochemical cues in harmony would better facilitate the myofibroblast differentiation *in vitro*. When these pre-differentiated myofibroblasts are transplanted at the wound site, these cells can further promote the remodeling of the damaged tissue^52^, primarily through the active production of matrix proteins. Therefore, in this study, we choose dermal fibroblasts and mimic three essential cues from the microenvironment, namely TGF-β1, polarized tension by aligned topography, and the electric field, to induce the differentiation of fibroblasts to myofibroblasts. Our study confirms the clinical potential of pre-tuned myofibroblasts as a new candidate for cell-based wound therapy.

## Materials and Methods

### Cell culture

Normal human dermal fibroblasts (NHDFs, ATCC, cell line) were cultured at 4000cell/cm^2^ density on the electrospun scaffolds. Cells were expanded for 2 days in Dulbecco’s Modified Eagle’s medium (DMEM, Lonza) supplemented with 10% fetal bovine serum (FBS, Lonza) and 1% penicillin-streptomycin (PS, Invitrogen). After cell expansion, cells were starved in the DMEM without FBS to maximize the efficacy of the subsequent chemical treatment on NHDFs. The differentiation of NHDFs to myofibroblasts was induced in DMEM supplemented with 10% FBS, 1% PS and 10 ng/ml of transforming growth factor-beta 1 (TGF-β1, Roche) for 2 days after 1 day of starvation in DMEM supplemented with 1% PS only. For all our experiments, only cells within 15 passage were used.

### Electrospinning

**Figure 1:**
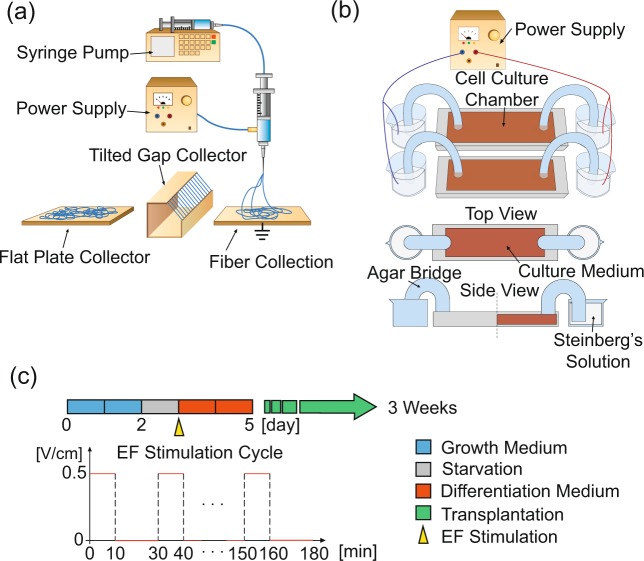
Illustration of experimental devices and design. (**a**) Electrospinning and fiber collectors to generate directionality on the scaffold. Tilted gap collector was used for fabricating aligned topography, and flat plate collector was used for random. (**b**) EF stimulation device for cell experiment. Voltage was given by the power supply and stimulated the cell indirectly through agar bridge to prevent electrophoresis in the culture medium. (**c**) Experimental cell culture condition and EF stimulation cycles.

20% w/v polycaprolactone (PCL, Sigma Aldrich) solution was used for electrospinning. The dichloromethane (DM, Junsei) and n, n-dimethylformamide (NDF, Junsei) were mixed at a 7:3 ratio and used as the solvent for the spinning process. The syringe pump was set to eject the PCL solution at 3 mL/h feed rate through a 25 G nozzle. For fabrication, 13 kV voltage was applied at the nozzle tip, and discharged fibers were collected on the grounded aluminum collector. The distance between the nozzle and the collector was 275 mm. The humidity of the lab was kept below 40%. The geometrical constraint of the collector administrated the directionality of electrospun fiber. In this report, we used both the flat plate and tilted gap collectors to generate the random and the aligned topography, respectively (Fig. 1(a)). When electrospun fibers are collected on a flat plate, they randomly dispersed forming a carpet of fibers without any directionality. In contrast, the tilted gap collector induced the asymmetric electric field between the plates, aligning the fibers^53^. The aligned electrospun fibers were then collected on a glass slide by simply adhering and stacking the fibers onto the surface of the glass.

### SEM Imaging

Electrospun fibers were coated with platinum using the sputter coater (Vacuum Device Inc.). The coating was initiated with 1 kV voltage for 30 s. Platinum coated fibers were observed at 10 kV accelerating voltages using scanning electron microscopy (SEM, FEI Company). Electron micrograph images were digitally recorded using xTm software (FEI Company) software based on which the directionality of the fibers was quantified.

### Scaffolds preparation

In order to ensure the handling convenience, a 1.5 × 1.5 cm^2^ square frame was fabricated around the edge of the electrospun sheet by squeezing the melted PCL through 20 G needle at 3 atm air using the direct polymer melting deposition (DPMD) method. The square scaffold was sterilized using a plasma generator for 45 s and immersed in ethanol for 24 hours under the ultraviolet light. All sterilized scaffolds were utilized within 12 hours.

### Electric field stimulation

NHDFs seeded scaffolds were placed in the stimulation chamber for expansion and starvation for 3 days, and on day 4, the EF was applied as the growth medium was replaced with the differentiation medium. The electric potential was generated by the power supply and indirectly stimulated the NHDFs through agar salt bridge immersed in the Steinberg’s solution (Fig. 1(b)). Steinberg’s solution, the pH buffer, was prepared by mixing 60 mM NaCl (Sigma Aldrich), 0.7 mM KCl (Sigma Aldrich), 0.8 mM MgSO_4_·7H_2_O (Sigma Aldrich), 0.3 mM Ca(NO_3_)_2_·4H_2_O (Sigma Aldrich) and 1.4 mM Tris base (Usb) in distilled water. The power source and the Steinberg’s solution reservoir was connected by a platinum wire. The reservoir was connected with the stimulation chamber through the agar salt bridge, made of 2% w/v agar powder in Steinberg’s solution. All parts of the EF devices were sterilized using autoclave before cell experiments. The condition for intermittent EF stimulation is schematically shown in (Fig. 1(c)). The EF was applied periodically as step pulses. Each cycle of EF was composed of 10 min-stimulation and 20 min-resting. Total stimulation duration was 3 hr, and the direction of EF was parallel to the aligned topography of electrospun scaffold. The magnitude of EF was 0.5 V/cm.

### Immunofluorescence

Before immunofluorescence, NHDFs sample was rinsed 3 times using 1X Dulbecco’s Phosphate-Buffered Saline (DPBS, Lonza) for 5 min each with scaffolds and prepared in the dried 6-well plate. Immunofluorescence was composed of 4 steps: fixation, permeabilization, blocking, and staining. All solutions in all steps were rinsed 3 times after treatment and placed dried surface in 6-well plate. Rinsed NHDFs were fixed immediately for 20 min using 3.7% (v/v) formaldehyde. After fixation, 0.2% (v/v) Triton-X (Sigma Aldrich) was added for 15 min permeabilization. Blocking process was done using 3% (w/v) BSA 2 times for 30 min each. During the staining step, we treat α-smooth muscle actin (α-SMA) antibody (Abcam) (with 1:100 dilution) for 12 hours, Alex Flour 488-fluorescence antibody (Invitrogen) (with 1:200 dilution) for 6 hours, Alex Fluor 568-phalloidin (Invitrogen) (with 1:50 dilution) for 20 min, and 4’,6-diamidino-2-phenylindole (DAPI, Molecular Probe) (with 1:50000 dilution) for 3 min. The multichannel fluorescence microscopy (Zeiss) was used for taking immunofluorescence images. Excitation/emission wavelengths of green, red, blue fluorescence were 495 nm/519 nm, 579 nm/604 nm, and 358 nm/461 nm, respectively.

### Intensity quantification for fluorescence images

**Figure 2:**
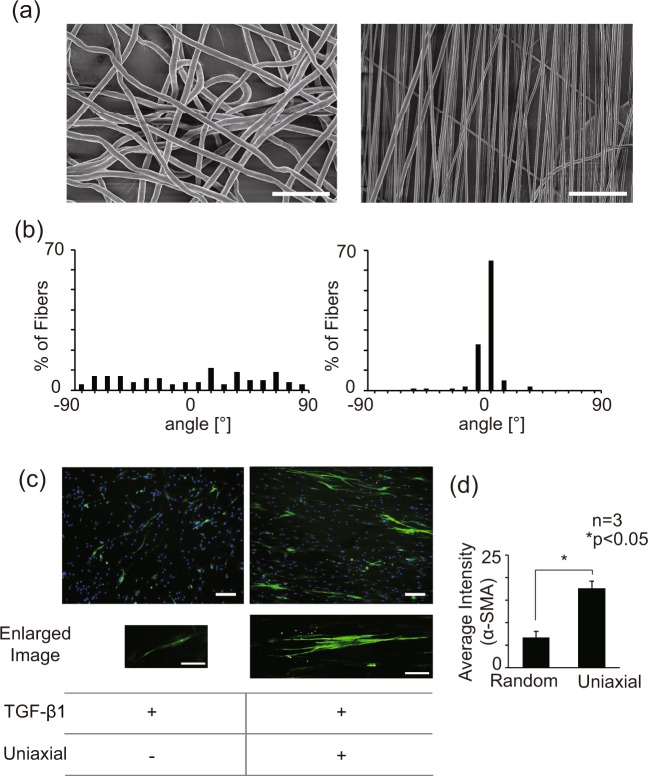
Characteristics of aligned fibers and the effects of aligned pattern on NHDF differentiation (**a**) SEM images of the random and aligned electrospun scaffold. (scale bar: 25 μm). (**b**) The angular distribution of random and aligned fibers. (**c**) Immunofluorescence images of NHDFs on random and aligned fiber with/without TGF-β1 treatment. (green: α-SMA, blue: nuclei, scale bar: 100 μm). (**d**) Green intensity (expression of α-SMA) graph of immunofluorescence images of NHDFs on random and aligned fiber. (Intensity pixel value: black-white, 0–255).

**Figure 3:**
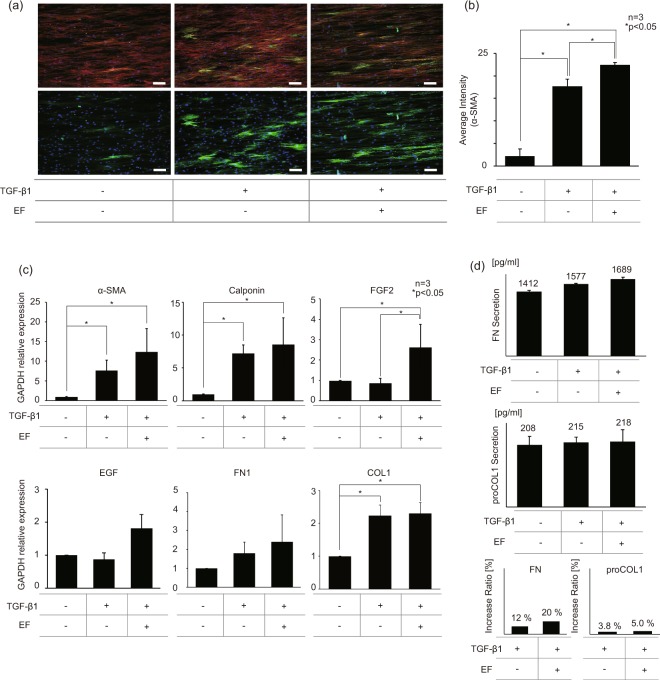
EF stimulation promotes NHDF differentiation. (**a**) Immunofluorescence images of NHDFs with/without EF. (red: actin green: α-SMA, blue: nuclei, scale bar: 100 μm). (**b**) green intensity (expression of α-SMA) graph of immunofluorescence images of NHDFs with/without EF. (intensity pixel value: black-white, 0–255) (**c**) qPCR results of NHDFs with/without EF. GAPDH was used as the housekeeping genes. The expression α-SMA, Calponin, FGF2, EGF, FN1, COL1 mRNA was quantified. (**d**) FN and proCOL1 ELISA results of NHDFs with/without EF. Amount of FN and proCOL1 secretion in culture medium were measured after the 2^nd^ day of differentiation period. The increase ratio of FN and proCOL1 secretion was calculated using ELISA results. The control group was NHDFs cells without any treatment.

All fluorescence images of GFP-tagged alpha smooth muscles were taken at 3 V with the exposure time of 300 ms. For average intensity quantification, shown in Figs 2(d) and 3(d), the images were first converted to grayscale, and the average intensity of the entire image window (1100 × 700 μm^2^) was measured using ImageJ software. Additionally, for image quantifying of collagen deposition in Masson’s Trichrome (MT) assay, all colors but blue color was subtracted by the “Select Color” function from Adobe Photoshop software. The blue color imaged were converted into grayscale followed by inverting of the images to visualize the collagen in white, and measured the average intensity using ImageJ software.

### qPCR

Before extracting mRNA, NHDF was washed 3 times with 1X DPBS. Added 700 μL Trizol (Takara) to both cell and scaffold, and gently mixed the solution by pipetting up and down until NHDF was dissolved entirely. The dissolved solution was collected in a 1.5 mL tube and vortexed for 5–10 seconds. After cell lysis, added 200 μL of chloroform (Sigma Aldrich), mixed for approximately 5 to 10 seconds with a vortex, and waited at room temperature for 1 minute until the mixture was separated into double layers. The layered solution was spun down at 4 °C, 12,000 rpm for 5 minutes to purify the mRNA. Isolated mRNA was collected from 400 μL from the top layer. Then, isopropanol (Merck) was mixed at a ratio of 1:1 and stored at room temperature for 20 minutes. The mRNA was spun down at 4 °C, 12,000 rpm for 10 minutes. All the solution except for the mRNA pellet was removed. The mRNA pellet was rinsed with 1 mL of 70% ethanol (Merck). After washing, all ethanol was removed, centrifuged for 5 minutes at 4 °C, 7500 rpm, and 20 μL of RNase-free water (Welgene) was added. The concentration of mRNA was quantitated using a spectrophotometer (Wilmington). cDNA synthesis was done using the iScripts TM kit (Bio-Rad). 1,000 ng of mRNA was mixed with 4 μL buffer and 1 μL of the reverse-transcribed mixture to synthesize the cDNA in 20 μL volume for each sample. Biometra T-personal Thermal Cycler was used for the cDNA synthesis with the following protocol: Initially started at 4 °C for 30 s, activated the primer binding for 5 min at 25 °C, incubated the cDNA reaction for 1 hour at 42 °C, and inactivated the enzymes for 5 min at 85 °C. Synthesized cDNA was stored at −20 °C. Real-time qPCR was performed using iQ SYBR green supermix (Bio-Rad). A Bio-Rad CFX96, real-time detection system, was used for the cDNA amplification first by initial denaturation at 95 °C for 5 min followed by 45 cycles of cDNA amplification (denaturation for 10 s at 95 °C, annealing for 30 s at 60 °C, an extension for 15 s at 72 °C). The gene expression levels were normalized to Glyceraldehyde 3-phosphate dehydrogenase (GAPDH) and calculated as the fold increase compared to the control. The following primers were used: GAPDH, α-smooth muscle actin (α-SMA), Calponin, Collagen Type I (COL1A1), Fibronectin (FN1), Epidermal Growth Factor (EGF), and Fibroblast Growth Factor (FGF2). The sequence of each primer was shown in Table 1.

**Table 1:** Primer sequence of NHDFs gene markers.

GAPDH	Forward: GGAGCGAGATCCCTCCAAAAT Reverse: GGCTGTTGTCATACTTCTCATGG
ACTA2	Forward: CTATGAGGGCTATGCCTTGCC Reverse: GCTCAGCAGTAGTAACGAAGGA
CNN1	Forward: GTCAACCCAAAATTGGCACCA Reverse: ACCTTGTTTCCTTTCGTCTTC
COL1	Forward: GAGGGCCAAGACGAAGACATC Reverse: GAGGGCCAAGACGAAGACATC
FN1	Forward: CGGTGGCTGTCAGTCAAAG Reverse: AAACCTCGGCTTCCTCCATAA
EGF	Forward: TGTCCACGCAATGTGTCTGAA Reverse: CATTATCGGGTGAGGAACAACC
FGF2	Forward: AGTGTGTGCTAACCGTTACCT Reverse: ACTGCCCAGTTCGTTTCAGTG

### Enzyme-linked immunosorbent assay (ELISA)

The sandwich ELISA employs anti-human fibronectin (FN, Takara) and procollagen type 1 (proCOL1, Takara) antibody coated in a microtiter plate. The proCOL1 ELISA was performed as follows: 100 μL of the antibody-POD conjugate solution and 20 μL of sample medium were transferred into the microtiter wells. The plate was allowed to incubate at 37 °C for 3 hours. After discarding the contents of the well, the plate was washed 4 times using wash buffer, and 100 μL of substrate solution was added for 15 min. The FN ELISA was done as follows: 100 μL sample medium was added into the microtiter wells for 1 hour at 37 °C and washed 3 times using wash buffer. Pipette 100 μL of antibody-POD conjugate solution for 1 hour at 37 °C. After that, cleaned 4 times and added 100 μL of substrate solution for 15 min and added 100 μL stop solution (1 N HCl, Sigma Aldrich). Both plates were analyzed right after the stop solution treatment. The developed color was measured using a microplate reader at a 450 nm wavelength.

### Animal model

The present study utilized immunodeficient Balb/c nude mice to suppress any immune responses to the transplanted human cells. All mice were anesthetized with intraperitoneal injection of 40 μL mixture containing rompun (40 mg/kg) and ketamine (10 mg/kg). Subsequently, a 12 mm excisional wound was cut on the middle of the dorsal surface by using a biopsy punch (Acuderm Inc., Fort Lauderdale, FL), and the differently conditioned scaffolds were transplanted onto the wound site, followed by covering with a transparent film (Opsite; Smith & Nephew, Andover, MA, USA) to prevent drying, detachment, and contamination. The transparent film is highly extensible and conformable with good moisture vapor permeability, allowing comfortable dressing during the entire experiment. We replaced the transparent film every 7 days to ensure better performance of the film. All experiments were approved by the animal care committee of Konkuk University (IACUC No. KU15151-1), and we performed the all animal experiment with the relevant guidelines and regulations of Konkuk IACUC.

### Histological analysis

For tissue staining, mice were sacrificed, and wound tissues were obtained after 7, 14, and 21 days. Specimens were fixed in 4% (v/v) paraformaldehyde (Sigma Aldrich), dehydrated with a graded ethanol series, and embedded in paraffin. The samples of 5 μm thickness were stained with hematoxylin and eosin (H&E) to measure immune response. Also, staining with Masson’s Trichrome (MT) was performed to assess the presence of collagen index in the wound regeneration tissues. After completing the steps of dehydration, samples on the glass slide were stained Bouin’s solution at RT for overnight, washed in the running water for 10 min. Then sections samples were incubated for 5 minutes with Weigert’s Iron Hematoxylin Solution. After Hematoxylin solution was discarded, the sections were incubated with Biebrich Scarlet-Acid Fuchsin Solution (Sigma-Aldrich) for 5 minutes and washed with the running tap water for 2 min. The sections were then sequentially incubated with Phosphotungstic/Phosphomolybdic Acid Solution (Sigma-Aldrich) and with the Aniline Blue Solution (Sigma-Aldrich) for 5 min each. Mounting with coverslips was performed using a histological mounting medium (national diagnostics, Atlanta, GA, USA) after rehydration.

### Statistical analysis

Statistical significance between mean values was determined by one-way analysis of variance (ANOVA) using GraphPad QuickCalcs. P-values ‘<0.05’ were considered statistically significant. All the error bars for the graphs indicate standard deviation.

## Results and Discussion

### Aligned topography accelerates the differentiation of NHDFs

TGF-β1, a tension inducer in dermal tissue, is known to play a critical role in the differentiation of fibroblasts to myofibroblasts^54,55^. The aligned topography of the cell culture platform has also been identified as a positive stimulant for fibroblast differentiation^56^. Here, we tested the combinatorial effects of these two physicochemical cues, namely TGF-β1 and aligned topography, on the differentiation of NHDFs to myofibroblasts. We cultured the NHDFs on aligned fibers in the culture media supplemented with 10 ng/ml of TGF-β1 to accelerate the myofibroblast differentiation. To achieve aligned topography in microscale, we utilized electrospinning of biocompatible PCL fibers to generate pseudo-3D fibrous mat of 30–50 μm. The uniaxial alignments of the fibers were induced by the asymmetric EF across the tilted gap collectors (Fig. 1(a)). The topographical features of random and aligned electrospun fibers were confirmed using SEM images (Fig. 2(a)). In random fibers, the angle distribution was broad with no preferential orientation.

In contrast, uniaxially spun fibers showed a narrow distribution where 88% of the fibers were within 10° of the reference axis (Fig. 2(b)). The average diameter of random and aligned fibers was 2.67 ± 0.47 μm and 1.10 ± 0.27 μm, respectively. Also, the porosity of each scaffold, defined as the area ratio of the void to the total mat, was measured using ImageJ software. The results indicate that porosity of the random and aligned scaffold was 37.18% and 38.89% with the apparent field to field variations, and the maximum pore sizes of the fiber mats were 295.0 μm^2^ and 374.8 μm^2^, respectively. As the typical average size of NHDFs is ~8000 μm^2^ with ~50, ~150 μm transverse/longitudinal axis length, the discrepancy in both the diameters and pore sizes between random and aligned fibers would have a negligible overall effect on the batch analysis.

The phenotypical changes of NHDFs, including morphology and differentiation state, were evaluated based on immunofluorescence images (Fig. 2(c)). Our results show that the aligned topography led to dramatic polarity changes in NHDFs. The directionality of the fibers guided the cell spreading by controlling the preference of the substrate attachment. Furthermore, we also confirmed that the aligned fibers promoted the differentiation of NHDFs to myofibroblasts (Fig. 2(d)). The differentiation level was quantified by measuring the intensity of green fluorescence, α-SMA, by ImageJ software. The average values of the pixel intensity of the entire images were 6.7 ± 1.4 and 17.7 ± 1.6 for the random and aligned condition, respectively. α-SMA expressions between random and aligned condition exhibited distinctive features. When cultured on randomly aligned fibers, most α-SMA expression was in the cytosol without any clear fibrous structures. On the other hand, the cells cultured on aligned scaffold showed thick fibrous α-SMA expressions over the entire cell, indicating the structural maturation of the NHDFs on the aligned topography. Although the detailed mechanism of promoting effects of aligned topography on NHDFs remains elusive, based on the fact that the high cellular polarity is known to correlate positively with the excess actin stress fibers^57,58^, it is plausible that NHDFs on aligned fibers produce more actin stress fibers to withstand their structural integrity, inducing the differentiation of NHDFs to myofibroblasts.

### Electric field promotes differentiation of NHDFs

Electrical stimulators have been used directly on the wound site as a therapeutic tool to recruit fibroblasts or to facilitate epithelial migrations^59–61^. In this study, instead of using the EF directly on the wound site, we utilized the intermittent EF of 0.5 V/cm for a total of 3 hours to pre-treat the fibroblasts for differentiation of fibroblasts to matured myofibroblasts. The effects of the EF on the NHDF differentiation were investigated using immunofluorescence technique (Fig. 3(a)). When the fluorescence images were converted to grayscale to measure the gray intensity values (0, 255), the average gray intensity of TGF- β1 treated NHDFs with/without EF, measured over three different fields, were 22.5 and 17.7, respectively (Fig. 3(b)).

The expression levels of mRNA and protein were measured using qPCR and ELISA to validate the fluorescence data quantitatively. The qPCR results indicate that TGF-β1 treated NHDFs had 7.7 times stronger expression of the most representative myofibroblast marker α-SMA compared to that of the untreated control, and EF stimulation additionally induced 12.4 times higher α-SMA gene expression compared to the sample without EF stimulation. Also, we observed the elevated expression of FGF2, one of the primary growth factors for fibroblast proliferation, at the mRNA level by adding EF stimulation (Fig. 3(c)). As for the ECM proteins, our ELISA results confirmed that the EF stimulation enhanced FN production by 8% more compared to the TGF-β1 treated counterpart with no EF. However, no significant difference in proCOL1 synthesis was detected in all conditions (Fig. 3d). Some discrepancy shown in the expression levels of genes and protein in the COL1 may be due to the temporal delay in the protein secretion following the gene regulations. Overall, we conclude that the dual stimulation by dcEF and TGF- β1 promoted the transformation of NHDF into further differentiated myofibroblasts, most likely representing distinctive functional cellular state compared to those of normal NHDF or TGF- β1 induced myofibroblasts.

### Myofibroblasts transplantation expedites the wound closing

**Figure 4:**
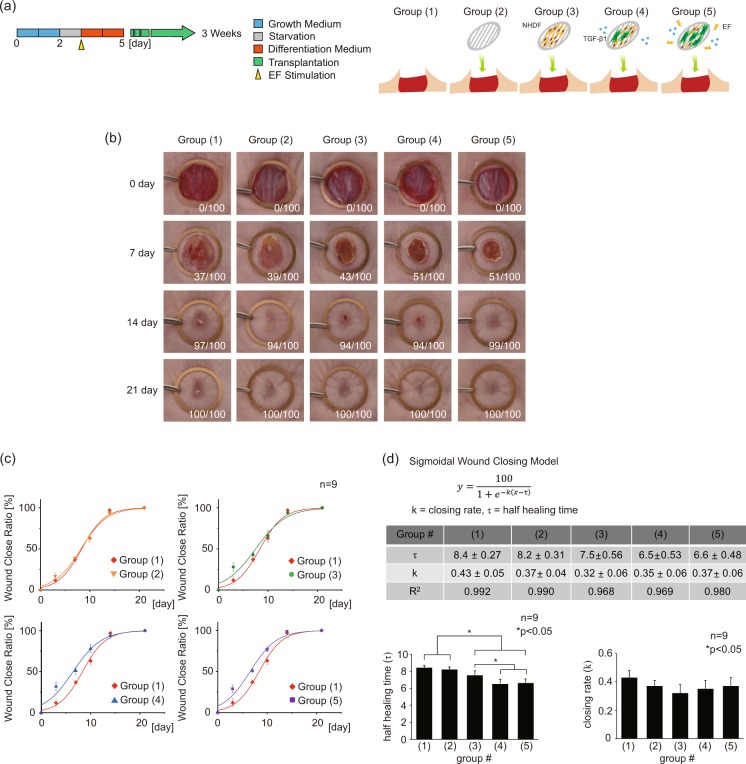
Wound closing ratio experiment in the mouse model. (**a**) Illustration of experimental Groups. The following five experimental Groups were studied: (1) natural healing (control), (2) bare scaffold, (3) scaffold with NHDFs, (4) scaffold with TGF-β1 treated NHDFs, (5) EF applied scaffold with TGF-β1 treated NHDFs. (**b**) Wound closing images for 0^th^, 3^rd^, 7^th^, 14^th^, 21^st^ day of healing. (**c**) The fitting curve graph of wound closing ratio. The standard sigmoidal function was applied to quantify the wound closing process. (n = 9). (**d**) The table and graph for variable values of the sigmoidal functions in each Group. Half healing time (τ, the time constant) and closing rate (k) were analyzed.

EF-induced myofibroblasts were transplanted on a mouse model with a 1.2 cm diameter punch wound to verify the feasibility of their uses for skin regeneration *in vivo*. The electrospun scaffolds with aligned fibers were utilized as the cell carrier. The scaffolds were fabricated to adequately cover the exposed wound on the back of a mouse. The changes in the wound diameter were tracked for 3 weeks, and the images of the wound closing were taken on 0^th^, 7^th^, 14^th^, and 21^st^ days to measure the wound closing rate (Fig. 4(a,b)). The following five experimental groups were studied: (1) natural healing (control), (2) bare scaffold, (3) scaffold with NHDFs, (4) scaffold with TGF-β1 treated NHDFs, (5) EF applied scaffold with TGF-β1 treated NHDFs. Images were quantified using ImageJ software. During the wound closing, the wound diameter progressively decreases until the wound is completely re-epithelialized by migrating epithelial cells. In Groups (1) and (2), the wound closing was measured to be only 37% and 39% on day 7, respectively. Likewise, Group (3) showed a slightly enhanced wound closing of 43%. The healed fraction of Group (4), which contained the myofibroblasts, reached up to 51%, possibly signifying the facilitation of the differentiated myofibroblasts for the faster closing of the wound *in vivo*. Group (5) exhibited a similar closing rate to that of Group (4).

We fitted the data points using the sigmoidal model, which is the most representative model for the natural wound closing (Fig. 4(c)). The average half healing time (τ), obtained from the sigmoidal curve fitting, showed a significant reduction in the Group (3), (4), and (5) by 0.9, 1.9, 1.8 days, respectively, compared to the Group (1). Furthermore, the Group (4) and (5) exhibited significantly faster half healing time compared to the Group (3), supporting a positive role of transplanted pre-conditioned myofibroblasts in the healing process. In contrast, the closing rate (k) showed no statistical difference between any of the groups despite the fact that there existed remarkably accelerated closure at day 3 in Groups (3), (4), and (5) (Fig. 4(d)). This discrepancy arises because the simple sigmoidal fitting fails to capture the accelerated healing at the earlier time; instead, the y-intercept at day 0 would be shifted upwards, leading to almost no change in the slope of the curve. When we calculated the initial slope of wound closing between day 0 and day 3 (k_0_), we noted significantly high k_0_ values in Groups (3), (4), and (5), (7.0, 8.0, and 7.3, respectively) compared to 4.0 and 4.3 for Groups 1 and 2, indicating the existence of non-sigmoidal behavior during the early healing phase. Based on this, it seems quite convincing to believe that the transplanted pre-conditioned fibroblasts have a transient effect on speeding up the early closure of the wound, which apparently does not provide direct influence on the epithelial cell migration for the later closure. However, the pre-conditioned fibroblasts in the Group (4) and (5) promoted the synthesis of the ECM proteins, accelerating earlier closure at the beginning. This has possibly led to better quality healing in these groups, as shown in Fig. 3(c).

To evaluate the implication of the accelerated healing that does not follow the sigmoidal growth profile, we employed a Weibull model to capture the effects of the pre-conditioned cells during the early stage of the healing process. M.A. Tabatabai *et al*. chose to use the Weibull model for the drug-assisted closing of the wound^62^. In this work, the wound of the Zn deprived mouse was treated with increasing Zn dosage to expedite the healing process, and the simple sigmoidal model failed to capture the initial rise in the closure rate. On the other hand, the Weibull-based hyperbolastic model successfully captured the drug-induced initial jump during the early stage of the healing. Similarly, we also observed initially enhanced closing rate on day 3 when the pre-conditioned cells were transplanted at the wound site. Based on this literature, we can deduce that the Weibull nature of the healing profile in Groups (3), (4), and (5) must originate from the difference between the control sample and the ones with transplanted cells (Fig. S1). This difference was obtained by subtracting the closing ratio of the control (natural healing) sample from each of the Groups to be compared. From the results, we inferred that the myofibroblasts in the Group (4) and (5) exhibited longer persistence (λ) of fast-initiation effects with large initial healing magnitude (M/λ).

### EF-stimulated myofibroblasts enhance the ECM protein synthesis at transplanted site

**Figure 5:**
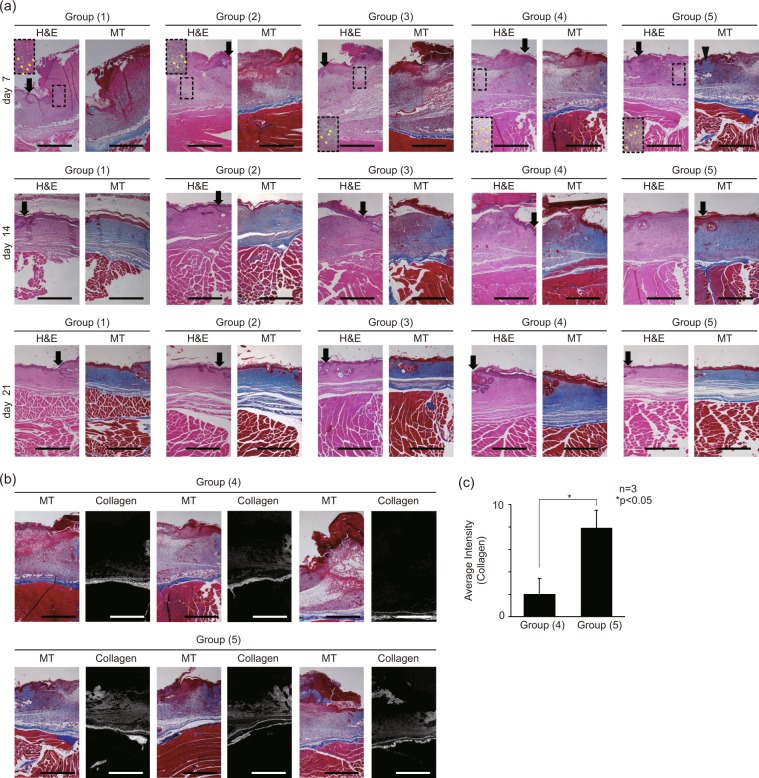
Tissue histology section images of mouse model wound sites. (**a**) Wound histology images for the 0^th^, 7^th^, 14^th^, 21^st^ day of healing. (black arrow: wound edge, yellow arrow: inflammation, black triangle: synthesized collagen) (**b**) MT assay images and the grayscale images of synthesized collagen (blue color) of MT assay images in the Group (4), (5). Three different mouse models were analyzed. (**c**) intensity (expression of α-SMA) graph of converted images to quantify the amount of collagen synthesized. (intensity pixel value: black-white, 0–255) The following five experimental Groups were studied: (1) natural healing (control), (2) bare scaffold, (3) scaffold with NHDFs, (4) scaffold with TGF-β1 treated NHDFs, (5) EF applied scaffold with TGF-β1 treated NHDFs.

The histology of the mouse wound model was evaluated to confirm the ECM deposition and overall regeneration effects of the wound patches containing cells of different conditions. Tissue sections from the following experimental Groups were prepared using H&E staining and MT assay: (1) natural healing (control), (2) electrospun scaffold, (3) scaffold with NHDFs, (4) scaffold with TGF-β1 treated NHDFs, (5) EF applied scaffold with TGF-β1 treated NHDFs(Fig. 4(a)). Figure 5(a) shows the representative images of H&E and MT assay from 3 sets of experiments. As shown, the wound healing histology was analyzed every 7 days for 3 weeks. The H&E staining images showed the inflammation process, marked by distinctive polymorphonuclear neutrophils, in all groups on day 7 (shown in yellow arrows), as well as the incomplete re-epithelization, represented by the uneven thickness of the epithelium, in all groups on day 7. During the typical wound healing process, the inflammatory stage proceeds the wound contraction, followed by matrix remodeling in the later stage. While the control sample in the Group (1) showed no sign of matrix regeneration by the 7^th^ day, both Group (4) and (5) clearly showed, the matrix remodeling, represented by newly synthesized collagen stained in blue at the sub-epidermal region, on the 7^th^ day along with the inflammatory traces. These results imply that the EF-stimulated myofibroblasts in Group (5) must have synthesized collagen even during the early stages of the repair process. Therefore, the myofibroblasts, which are known to have outstanding ECM production capability^63^, likely have promoted the healing process by encouraging the synthesis of matrix proteins essential for faster closure of the wound. To quantify the collagen synthesis from the MT assay images on day 7 of the Group (4) and (5), only blue was extracted from each image in Fig 5(b). From these images, we verified that the EF simulated myofibroblasts in the Group (5) dramatically enhanced the collagen synthesis at the wound sites in all three different mouse models. The average intensity of the pixel values for synthesized collagen in the Group (4), (5) were 2.0 and 7.9, respectively (Fig. 5(c)).

Furthermore, this elevated collagen deposition, shown in the Group (4) and (5), could have led to rapid re-epithelization shown on the 14^th^ day of wound healing. The H&E staining of the Group (4) exhibited the irregularity in the epithelial thickness, implying the imperfect closure of the epithelium. In contrast, the stratified and ordered epithelium in the Group (5) must have been achieved by the enhanced re-epithelization process, provoked by fast matrix regeneration. As for the collagen synthesis, the *in vivo* histological measurements were not in perfect agreement with either the *in vitro* qPCR or ELISA results (Fig. 3(c,d)). Despite the apparent difference in collagen distribution and synthesis between Group (4) and (5) shown in the histological results, the *in vitro* data of these two groups were not significantly different. However, we must remind ourselves of the fact that the myofibroblasts of the two different experimental groups, namely ones differentiated by TGF-β1 only and the other differentiated by TGF-β1 and EF, exhibited distinctive functional cellular states featuring differential gene and protein expressions (Fig. 3(c,d)). Thus, it is plausible that the myofibroblasts differentiated by dual cues would behave differently to ones differentiated by TGF-β1 only when transplanted at the wound sites, induced to better synthesize collagen or to promote the neighboring cells to synthesize collagen *in vivo*. However, excessive collagen deposition may lead to fibrotic healing with impaired regeneration of hair follicles and dermal glands. Therefore, depending on the severity of the wound, the number of transplanted myofibroblasts must be optimized in case this strategy were to be applied in human patients in the future.

## Conclusion

This study aims to suggest a strategic approach to enhance the effectiveness of the cell transplantation for the wound regeneration process. Instead of utilizing stimulating devices or substances directly to the wound site or on the wound dressing, we propose a novel strategy of pre-treating the cellular constituents prior to the transplantation such that the already pre-tuned cells can better enhance the healing process.

Skin tissue consists of many cell types, including keratinocytes, fibroblasts, myofibroblasts, endothelial cells, and immune cells, each of which serves specific functions during the regeneration process. For example, the keratinocytes in epidermis participate in wound closing process, whereas the fibroblasts in the dermis contribute to matrix reconstruction. Therefore, selecting appropriate cell types is essential for developing the engineered skin depending on the specific conditions of the wound^64,65^. In this paper, we propose to utilize functionally suitable myofibroblasts that have been pre-conditioned by dual physicochemical cues as the candidate cellular constituents in skin tissue engineering. Our research findings suggest that the simultaneous application of TGF-β1 and EF with aligned micro-environment significantly increased the differentiation of fibroblasts to myofibroblasts, and upregulated a few essential healing-related genes and proteins. The results of the animal experiment may suggest the existence of paracrine effects of the physicochemically tuned myofibroblasts on the neighboring cells in the transplanted site, promoting matrix remodeling in the vicinity.

For this reason, we suggest that supplementing the EF to the generic TGF-β1 treatment would be strategic in generating activated myofibroblasts, and the pre-activated myofibroblasts can be a great candidate for the engineered tissue substitute. In physiological tissue repair, myofibroblasts either naturally disappear through apoptosis or become dedifferentiated into fibroblasts as the healing progresses. When these myofibroblasts undesirably persist in a closed wound, a hypertrophic scar may be induced. Although the actual fate of the transplanted myofibroblasts used in our study has not been followed, any fibrotic consequences should be carefully considered in further studies.

For severe wounds, many experimental attempts and clinical trials have utilized both chemical and physical treatments directly on the wound sites. However, the condition of the direct application of such treatments would depend sensitively on the severity, size, and shape of the wound. In contrast, the utilization of cells that have been pre-treated by physicochemical stimuli would have definite advantages of being less sensitive to the specific conditions of the wound. Although the detailed mechanism of how the dual physicochemical cues of TGF-β1 and EF synergistically differentiate the NHDFs to the myofibroblasts is not clear yet, the phenomenological outcomes from this study can be inferred in utilizing the EF stimulation on various tissue engineering applications.

## Supplementary information

Figure S1
