# Supplementary material for: Physicochemically Tuned Myofibroblasts for Wound Healing Strategy
Source: Sci Rep. 2019 Nov 5;9:16070. doi: 10.1038/s41598-019-52523-9 (PMC6831678; doi:10.1038/s41598-019-52523-9)
Supplement: Supplementary file 1 — Figure S1 [file 41598_2019_52523_MOESM1_ESM.docx]

**Physicochemically Tuned Myofibroblasts for Wound Healing Strategy**

Ung Hyun Ko^1^, Jongjin Choi^2, 3^, Jinseung Choung^1^, Sunghwan Moon^2*^ and Jennifer H. Shin^1*^

^1^ Department of Mechanical Engineering, Korea Advanced Institute of Science and Technology, Daejeon, Republic of Korea

^2^ School of Medicine, Konkuk University, Seoul, Republic of Korea

^3^ BYON Co. Ltd., Seoul, Republic of Korea

^*^Corresponding Authors:

Sunghwan Moon:

- Tel: +82-2-2049-6028

- E-mail Address: sunghwanmoon@kku.ac.kr

- Postal Address: Konkuk University, Industrial Alliance Building Room 319, 120 Neungdong-ro, Gwangjin-gu, Seoul 05029, Republic of Korea

Jennifer H. Shin:

- Tel: +82-42-350-3232

- E-mail Address: j_shin@kaist.ac.kr

- Postal Address: Korea Advanced Institute of Science and Technology, Mechanical Engineering Building Room 2215, 291 Daehak-ro, Yuseong-gu, Daejeon 34141, Republic of Korea

**Keywords:** Skin Tissue Engineering, Wound Healing, Myofibroblast, Electric Field, Aligned Topography

**
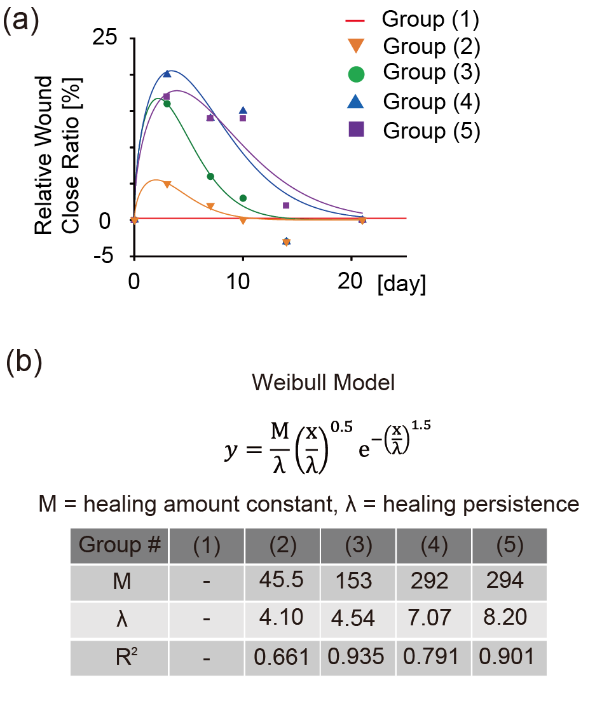
**

**Figure S1.** Weibull model fitting for the difference in the wound closing profile. The relative wound closing ratio was obtained by subtracting the closing ratio of the control (natural healing) sample from each of the Groups to be compared. (a) The fitting curve graph of Weibull model for the difference in closing rate. (n=9) (b) The tables for variable values of the Weibull parameters in each Group. M (healing amount constant), λ (healing persistence) were calculated. The following five experimental Groups were studied: (1) natural healing (control), (2) bare scaffold, (3) scaffold with NHDFs, (4) scaffold with TGF-β1 treated NHDFs, (5) EF applied scaffold with TGF-β1 treated NHDFs.
